# Effects of minimally invasive surgery combined with specialized pain management nursing care on postoperativepain improvement and life quality after spinal injury

**DOI:** 10.12669/pjms.40.6.8652

**Published:** 2024-07

**Authors:** Na Zhong, Hai-yun Wang, Qiong Wei, Hui Li, Na Zhang, Jian-xue Hao

**Affiliations:** 1 Na Zhong Department of Orthopaedics, Baoding No.1 Hospital, Baoding, Hebei, 071000, P.R. China; 2 Hai-yun Wang Department of Orthopaedics, Baoding No.1 Hospital, Baoding, Hebei, 071000, P.R. China; 3 Qiong Wei Department of Orthopaedics, Baoding No.1 Hospital, Baoding, Hebei, 071000, P.R. China; 4 Hui Li Department of Orthopaedics, Baoding No.1 Hospital, Baoding, Hebei, 071000, P.R. China; 5 Na Zhang Department of Orthopaedics, Baoding No.1 Hospital, Baoding, Hebei, 071000, P.R. China; 6 Jian-xue Hao Department of Orthopaedics, Baoding No.1 Hospital, Baoding, Hebei, 071000, P.R. China

**Keywords:** Specialized Pain Management Nursing Care, Spinal injury, Minimally invasive surgery, Pain, Quality of life

## Abstract

**Objective:**

To determine the impacts to research the impacts of pain’s Specialized Pain Management Nursing Care in the perioperative period on pain symptoms and life quality of patients experiencing minimally invasive surgery for spinal injury.

**Method:**

Eighty patients with a spinal injury who underwent minimally invasive surgery in the Department of Orthopedics of Baoding No.1 Hospital from January 2018 to December 2021 were retrospectively analyzed. They were split into two groups following different nursing methods (n=40 each group). Specialized Pain Management Nursing Care were given to patients in the observation group. Those in the control group were given treated with routine care. Their pain score and nursing effect were compared, after which their quality of life, daily living ability and complication rate compared and analyzed.

**Results:**

The pain degree in the control group was considerably more than that in the observation group in the 1st postoperative period. The pain degree, which decreased in both groups, slumped more significantly in the observation group on the 2nd and 3rd postoperative days. The postoperative hospital stays and pain duration in the observation group were shorter than those in the control group (*P*<0.05), and the nursing effect was significantly better than that in the control group (*P*<0.05). After postoperative nursing intervention.

**Conclusion:**

Minimally invasive surgery integrated with the Specialized Pain Management Nursing Care can remarkably ameliorate pain after spinal injury surgery, reducing complications’ incidence, and improving the life quality for patients.

## INTRODUCTION

Under the interaction of a variety of factors, spinal injury seriously affects patients’ health and normal work and life. Once the onset of spinal injury, it will undermine patients’ mobility and body function severely, and in severe cases threatens the life safety of patients. For this reason, surgery is the preferred clinical method for spinal injury, it possesses the following benefits: less trauma, high safety, less pain, fewer complications, more efficient postoperative recovery and shorter hospitalization.^1^,^2^ Surgery led to a series of adverse consequences, including trauma, increased pain, physical and mental pain, affecting the prognosis of patients and lowering their life quality.

Patients experiencing spinal injuries are affected by numerous factors regarding their postoperative quality of life, of which postoperative pain is a crucial factor. Pain has been broadly recognized as the fifth vital sign, and it will respond to the patient’s psychological, physical and behavioral aspects, it may turn into chronic pain that not only affects the success or failure of surgery, but also has an immeasurable impact on patient outcomes and quality of life.^3^,^4^ Consequently, active and effective nursing interventions should be taken clinically to relieve pain, ameliorating clinical prognosis, promoting patients’ early recovery and improving their life quality. Based on this, in this study, a detailed exploratory analysis was conducted on the impact of minimally invasive surgery combined with the Specialized Pain Management Nursing Care on the pain degree and patients’ life quality after spinal injury.

## METHODS

This is a retrospective study. Eighty patients with a spinal injury who had undergone minimally invasive surgery in the Department of Orthopedics of Baoding No.1 Hospital from January 2018 to December 2021 were analyzed retrospectively. They were split into two groups following different nursing methods: the observation and control group. There were forty cases per group.

### Ethical Approval

The study was approved by the Institutional Ethics Committee of Baoding No.1 Hospital (No.:2021036; date: March 26, 2021), and written informed consent was obtained from all participants.

### Inclusion criteria:

- Patients who were clinically diagnosed with spinal injury and had undergone minimally invasive surgery.^5^
- Patients whose records were completed to show completion of follow up.
- Patients who were able to re-examine on time after surgery and had complete follow-up data.
- Patients who themselves and their family members took part in this research and signed the informed permission.

### Exclusion criteria:

- Patients who had ankylosing spondylitis.
- Patients who had fractures in other parts or obvious pain caused by other diseases.
- Patients with a malignant tumor, chronic inflammatory conditions and dysfunction of important organs.
- Patients with incomplete clinical data.

Regular clinical nursing was received by patients in the control group, including health education, psychological guidance and medication guidance, etc. The observation group enderwent specialized pain care in addition to usual care.

The observation group underwent specialized pain care in addition to usual care. Routine was given on the basis of everyday nursing in the control group. In the observation group, Specialized Pain Management Nursing Care was given in addition to regular nursing.

- Preoperative: patients were assisted to consummate in preoperative examination, guided to perform adaptive training, and build a satisfactory nurse-patient tie.
- Intraoperative: Patients were closely monitored for vital signs, fluids were replenished in time, and limb micro-movements were assisted
- Postoperative: All vital signs were recoded and drainage tube was looked after. If any abnormality was found, feedback was given in time to deal with it. Wound dressing contamination was replaced in time to prevent infection.

### Specialized Pain Management Nursing Care:

Patients were given an explanation of the precautions and the relationship between pain and disease preoperatively to increase their confidence and determination to overcome pain. Adaptive training was conducted for patients to guide them to master the correct breathing method and avoid the phenomenon of aggravated pain caused by improper breathing. Postural training was conducted to guide patients to master the correct postural placement postoperatively.

### Analgesic intervention

Analgesic intervention was carried out for patients, analgesic drugs were adjusted in time according to the pain degree, pain changes of patients were closely observed, and medication was guided by the three-level analgesic method.

### Position guidance

Supine position was the routine position after surgery. To avoid the front and back, left and right displacement of the spine, the position was placed as far as possible according to the physiological radian of the spine. When fixing limbs and changing dresses, patients were assisted to place limbs correctly to avoid pain.

Visual analog scale (VAS) was utilized to determine the pain level of the two groups preoperatively. Simultaneously, counted the span of hospitalization time and postoperative pain. The nursing effect was evaluated based on the relevant indicators in the “Guidelines for Diagnosis and Treatment of Orthopedic Diseases in China”. Meanwhile, the quality of life (QOL-C30) levels and activities of daily living (ADL) of the two groups were compared: QOL was evaluated by the quality-of-life scale (QLQ-C30), including four facets: cognitive function, physical function, role function, and social function. Each had a maximum of 100 points. The higher the score, the better the life quality; ADL was assessed by the modified BI index, which included 10 items such as eating, bathing, dressing, etc. Each item was scored according to whether help was needed and the degree of help needed. The maximal score was 100: the higher the score, the more substantial the ability. Postoperative complications were recorded and compared between both groups.

### Statistical analyses

SPSS22.0 software was utilized to compare data in this research, and measurement data were presented as (*χ̅*±*S*). Mean ± standard deviations were calculated for continuous variables. For inter-group comparison test was adopted. Paired T-test was employed for intra-group mean comparison before and after treatment. Chi-square test was also performed to analyses categorical variables. Results were considered significant at p<0.05.

## RESULTS

The observation group included 22 males and 18 females aged 23-71. Their average age was (46.88±11.43) years. The control group included 24 males and aged 25-70, with an average age of (47.23±11.74) years. No statistically substantial contrast was detected in the elementary data between both groups, which were comparable (P > 0.05), as showed in Table-I.

**Table-I:** General data comparison between both groups.

Item	Observation group (n=40)	Control group (n=40)	t/² value	P value
Age	46.88±11.43	47.23±11.74	-0.135	0.893
Gender (M/F, n)	22/18	24/16	0.205	0.651
Disease type (n)			7.521	0.057
Spinal stenosis	6	5		
Lumbar disc herniation	3	10		
Spinal fractures	18	20		
Spinal deformities	13	5		

There was no significant difference in preoperative pain degree between the both groups. On the first postoperative day, the pain degree of both groups was significantly higher than the preoperative period. The pain degree of the observation group was meaningfully lower than the control group (p<0.05). On the 2^nd^ and 3^rd^ postoperative days, the pain level in both groups lowered. However, the observation group slumped more sharply in pain level. The postoperative hospitalization and pain duration in the observation group were shorter than the control group, with statistically considerable differences (*P*<0.05), as demonstrated in Table-II. After surgery, the nursing effect of patients in the observation group was significantly better than that in the control group, with a statistically significant difference (P<0.05), as shown in Table-III.

**Table-II:** Comparison of VAS score, span of hospitalization and pain duration between both groups at different postoperative periods (*χ̅*±*S*).

Group	VAS score				Length of hospital stay (d)	Postoperative pain duration (d)

	Before surgery	1d after surgery	2d after surgery	3d after surgery		
Observation group (n=40)	2.60±0.93	5.70±0.85	2.80±0.65	1.43±0.50	10.30±1.22	5.38±0.70
Control group (n=40)	2.58±0.81	6.50±0.68	3.50±0.60	1.93±0.80	12.80±1.20	6.83±0.71
T value	0.128	-4.639	-5.014	-3.360	-9.216	-9.153
P value	0.898	0.000	0.000	0.001	0.000	0.000

**Table-III:** Comparison of nursing effect between both groups (%).

	Markedly effective	Effective	Invalid	Effective rat
Observation group (n=40)	23 (57.50)	15 (37.50)	2 (5.00)	38 (95.00)
Control group (n=40)	17 (42.50)	13 (32.50)	10 (25.00)	30 (75.00)
*χ*^2^ value				6.275
*P* value				0.012

There was no significant difference in preoperative QOL and ADL scores between the two groups (p>0.05). After nursing intervention, the QOL score and ADL score of patients in the two groups were significantly improved, but the QOL score and ADL score of patients in the observation group were significantly higher than those in the control group (p<0.05), Table-IV.

**Table-IV:** Comparison of QOL and ADL scores between the two groups prior to and later than intervention (*χ̅*±*S*).

Group	QOL score				ADL score			

	Before intervention	After intervention	T value	P value	Before intervention	After intervention	t value	P value
Observation group (n=40)	55.65±4.94	85.55±4.81	-27.420	0.000	44.60±3.78	75.35±3.93	-35.649	0.000
Control group (n=40)	54.85±4.70	76.95±4.49	-21.495	0.000	43.80±3.65	61.95±3.73	-21.995	0.000
t value	0.742	8.258			0.962	15.641		
P value	0.460	0.000			0.339	0.000		

Postoperative complications occurred in both groups, among which the incidence of phlebitis was the highest, and the total incidence of patients in the observation group was lower than that in the control group, with a statistically significant difference (P<0.05), as shown in Table-V.

**Table-V:** Comparison of postoperative complications between both groups (%).

	Pressure score	Phlebitis	Lung infection	Urinary tract infection	Wound infection	Total incidence
Observation group (n=40)	0 (0.00)	2 (5.00)	1 (2.50)	2 (5.00)	1 (2.50)	6 (15.00)
Control group (n=40)	1 (2.50)	5 (12.50)	2 (5.00)	3 (10.00)	3 (7.50)	14 (35.00)
χ^2^ value						4.267
P value						0.039

## DISSCUSSION

In this research, the degree of pain in the observation group was lighter than that in the control group on the 1st, 2nd and 3rd day after operation, and the duration of hospitalization and postoperative pain was shorter than that in the control group(p<0.05). The nursing effect was better than that in the control group(p<0.05).

Postoperative complications in the observation group was lower than that in the control group(p<0.05). The reason may be that special nursing care for pain has strengthened the perioperative precautions of patients and reduced the incidence of complications; Moreover, the reduction of pain degree can enhance the metabolic status of patients and reduce the occurrence of pain stress reaction during perioperative period. Most patients after spinal surgery have difficulty falling asleep due to severe pain, which reduces the quality of sleep. This not only prolongs the recovery period of patients after surgery, but also causes various postoperative complications.

Spinal surgery has a large trauma, a lot of bleeding during the operation, and severe pain after the operation, as a result patients are afraid of surgery.. In addition, pain can cause patients to have negative emotions and irritability.^6^,^7^ The reduction of treatment compliance will lead to the reduction of clinical operation effect, delay of postoperative rehabilitation, and even affect the quality of life of patients. In order to help patients reduce postoperative pain, it is necessary to implement special nursing. Special pain care can help patients increase their confidence in overcoming diseases, reduce the adverse effects of pain, increase the satisfaction rate of clinical treatment and improve the quality of life of patients after surgery by strengthening psychological care for patients and increasing pain health education.^8^

Therefore, appropriate perioperative nursing has a great impact on the clinical treatment of patients.^9^,^10^ Special pain nursing is an intervention for postoperative pain, aiming at alleviating pain stimulation, improving patient comfort, and promoting rapid recovery of patients. It can significantly relieve pain, improve patients’ anxiety and negative emotions during perioperative period, and improve patients’ quality of life during perioperative period.^11^-^13^

Special pain care can reduce the postoperative pain of patients, enable patients to perform early rehabilitation exercises, and reduce the occurrence of complications.^14^,^15^ In addition, effective pain intervention during peripheral surgery can also alleviate and eliminate the adverse effects caused by pain, such as anxiety, irritability, neurasthenia, etc., which can effectively improve the quality of life and daily living ability of patients.^16^,^17^ In this study, the quality of life and ability of life of patients in the two groups were significantly improved after surgery. The improvement of the above two indicators in the observation group who received special pain care was higher than that in the control group (p<0.05).

### Limitations

It is a retrospective study and the sample size is small. Thus, the curative effect of the technology is to be further verified via long-term randomized control studies.

## CONCLUSION

In the process of minimally invasive treatment of spinal injury, special care for pain can reduce the degree of postoperative pain, reduce the duration of pain, the length of hospitalization, the incidence of complications, and improve the patient’s satisfaction with surgery, daily living ability and quality of life.

### Authors’ Contributions:

**NZ** and **HW:** Carried out the studies, collected data, and drafted the manuscript, and are responsible accountable for the accuracy and integrity of the work.

**QW** and **HL:** Performed the statistical analysis and participated in its design.

**NZ:** and **JH:** Participated in acquisition, analysis, or interpretation of data and drafted the manuscript. All authors read and approved the final manuscript.
